# Temporal regulation of proteome profile in the fruit fly, *Drosophila melanogaster*

**DOI:** 10.7717/peerj.2080

**Published:** 2016-05-24

**Authors:** Perumal Subramanian, Jaime J. Jayapalan, Puteri S. Abdul-Rahman, Manjula Arumugam, Onn H. Hashim

**Affiliations:** 1 Department of Biochemistry and Biotechnology, Annamalai University, Chidambaram, Tamil Nadu, India; 2 University of Malaya Centre for Proteomics Research (UMCPR), Faculty of Medicine, University of Malaya, Kuala Lumpur, Malaysia; 3 University of Malaya Centre for Proteomics Research (UMCPR), Department of Molecular Medicine, Faculty of Medicine, University of Malaya, Kuala Lumpur, Malaysia

**Keywords:** Proteome, *Drosophila melanogaster*, Circadian, Metabolism, Mass spectrometry

## Abstract

**Background.** Diurnal rhythms of protein synthesis controlled by the biological clock underlie the rhythmic physiology in the fruit fly, *Drosophila melanogaster*. In this study, we conducted a proteome-wide investigation of rhythmic protein accumulation in *D. melanogaster*.

**Materials and Methods.** Total protein collected from fly samples harvested at 4 h intervals over the 24 h period were subjected to two-dimensional gel electrophoresis, trypsin digestion and MS/MS analysis. Protein spots/clusters were identified with MASCOT search engine and Swiss-Prot database. Expression of proteins was documented as percentage of volume contribution using the Image Master 2D Platinum software.

**Results.** A total of 124 protein spots/clusters were identified using MS/MS analysis. Significant variation in the expression of 88 proteins over the 24-h period was observed. A relatively higher number of proteins was upregulated during the night compared to the daytime. The complexity of temporal regulation of the *D. melanogaster* proteome was further reflected from functional annotations of the differently expressed proteins, with those that were upregulated at night being restricted to the heat shock proteins and proteins involved in metabolism, muscle activity, protein synthesis/folding/degradation and apoptosis, whilst those that were overexpressed in the daytime were apparently involved in metabolism, muscle activity, ion-channel/cellular transport, protein synthesis/folding/degradation, redox homeostasis, development and transcription.

**Conclusion.** Our data suggests that a wide range of proteins synthesized by the fruit fly, *D. melanogaster*, is under the regulation of the biological clock.

## Introduction

A broad spectrum of physiological, cellular, biochemical, endocrinological and molecular functions in living systems display temporal 24 h rhythms. As a consequence, the extent of biological processes regulated by biological clock vary from sleep-wake patterns, body temperature, activities of numerous enzymes, hormones, synthesis of nucleic acids and cell division (Moore-Ede, Sulzman & Fuller, 1982). As various aspects of cell metabolism and cell division cycle are regulated by biological clock (Asher & Schibler, 2011; Bass & Takahashi, 2010) it could be easily hypothesized that proteome profile of a living organism could be circadian in nature.

Previous studies showed that 30% of mRNA transcripts exhibit circadian variation (Lück et al., 2014). The circadian regulation of posttranslational processes has also been revealed by proteomic studies of the circadian rhythm (Robles, Cox & Mann, 2014) and studies on the variation of the cerebrospinal fluid over the light-dark cycle have been reported (Teixeira-Gomes et al., 2015). Earlier, the global level of circadian proteome of whole mouse liver (inclusive of various functional parts—left and right triangular ligaments, fissure for ligamentum teres, fissure for ligamentum venosum, hepatic veins, etc.,) has been investigated by Reddy et al. (2006). This report revealed a contrasting variation of protein profile between day and night. Whilst many genes have been demonstrated to coordinate rhythms in RNA synthesis, splicing and translation, numerous others also exhibited significant temporal disconnections between these functions (reviewed in Beckwith & Yanovsky, 2014). Hence, circadian oscillations represent a perfect system to comprehend how manifold transcriptional and post-translational processes are integrated rhythmically to maximize the fine-tuning of functions of organisms to the environment cycle.

Although rigorous research has been carried out on molecular genetics and developmental studies in the fruit fly, *Drosophila melanogaster*, little is known about the proteome of the fly. Proteomics is a central aspect in systems biology of the fruit fly that appends a distinctive dimension in investigating gene function and regulatory mechanisms. Hence, the temporal pattern of proteome profile would add useful information for further research and analysis. Search of PubMed (http://www.ncbi.nlm.nih.gov/pubmed/) references illustrated that by the end of October 2015 articles on *Drosophila* proteomics constitute only 0.95% of all published papers on proteomics.

The circadian clock is conventionally thought to exist in the lateral neurons of the fly brain (Kaneko, Helfrich-Forster & Hall, 1997). Intriguingly, brain-independent circadian oscillations have been perceived in almost all peripheral tissues of *D. melanogaster*. For instance, the same circadian rhythm of *β*-galactosidase expression has been demonstrated in the Malpighian tubules of decapitated and non-decapitated flies bearing the *per-lacZ* reporter transgene (Hege et al., 1997). In addition, Plautz et al. (1997a) has demonstrated the appearance, disappearance and reappearance of PERIOD protein in a rhythmic (24 h) pattern in the head, abdomen, thorax, legs and wings of the fly, indicating that numerous cellular processes all over the body of the fly are regulated by the temporally oscillating circadian clock gene, *period*. It is in this context, the present study has been carried out.

Currently, very little has been documented about the rhythmic build-up of proteins in *D. melanogaster* (Mauvoisin et al., 2014). To investigate the overall circadian transcriptional regulation in *D. melanogaster*, Rodriguez et al. (2013) separated nascent RNA from fly heads at six time points over a 24 h period (00:00, 04:00, 08:00, 12:00, 16:00 and 20:00 in 12:12 h light-dark cycle) and their data specified a key role of posttranscriptional control to fly’s circadian mRNA oscillation. Following this temporal schedule, we have investigated the overall pattern of temporal proteome (i) to complement the available data in fly literature, and (ii) to reveal the integrated pattern/regulation of circadian proteome in the whole body of the fly.

## Materials and Methods

### *Drosophila* culture and sample collection

*D. melanogaster* (wild type-Canton S) flies were maintained on medium comprising maize powder, sucrose, yeast and nepagin (anti-fungal agent) at 21 ± 2 °C under 12 h:12 h (light:dark) phases. We have used the whole fly for the proteomic study as performed in typical proteotypic peptide (PTP) studies reported earlier (Brunner et al., 2007) and followed the same protocol with trypsin digestion. The adult male flies (seven days old) were collected at 4 h intervals (Rodriguez et al., 2013) over a 24 h period (at 00:00, 04:00, 08:00, 12:00, 16:00 and 20:00). The flies collected at each time point (*n* = 15) were suspended in sample solubilization solution (100 µL) containing equal volume of SDS (1%) and *β*-mercaptoethanol (5%) and were swiftly frozen in liquid nitrogen. The flies were homogenized and the homogenate was kept at −80 °C until analysis. The proteins were solubilized at 95 °C in sample solubilization solution and vortexed. The miniature cuticle residues were sedimented by centrifugation at 8,200 g (5 min). The solubilized proteins were precipitated in TCA (20%) in cold acetone (90%) along with dithiothreitol (DTT, 20 mM) on ice (Jessie, Hashim & Rahim, 2008).

### Assay for protein estimation

The total protein content of fly homogenate was estimated (Bradford, 1976) after pre-treatment and re-solubilization of protein pellet with sodium hydroxide (0.2 M) and rehydration buffer (urea (7 M), thiourea (2 M), CHAPS (4%, 3-[(3-cholamidopropyl) dimethylammonio]-1-propanesulfonate) and bromophenol blue (0.002%)), respectively, as previously described (Jessie, Hashim & Rahim, 2008).

### 2-D gel electrophoresis (2DE) and silver staining

2DE was performed with 50 µg of precipitated (TCA/acetone) fly proteins. The proteins were incubated in rehydration buffer (urea (7 M), thiourea (2 M), CHAPS (4%), IPG buffer (pH 3–10 NL), DTT (65 mM) and bromophenol blue (0.002%)) at 25 °C for about 12 h and loaded onto 13 cm rehydrated precast immobilized dry strips (pH 3–10 non-linear, GE Healthcare BioSciences, Uppsala, Sweden). The strips were subjected to isoelectric focusing with EttanIPGhor 3 Isoelectric Focusing Unit (GE Healthcare, Uppsala, Sweden) for a total time of 20 kV/h. Focused strips were equilibrated in Tris–HCl (1.5 M, pH 8.8 with urea (6 M), SDS (2%), glycerol (30%) and DTT (0.06 M)) for 20 min and subsequently incubated in a similar equilibration solution but containing iodoacetamide (4.5%) in lieu of DTT for 20 min. The equilibrated strips were then overlaid onto homogenous polyacrylamide gel (12.5%) and electrophoresis was carried using the SE 600 Ruby Electrophoresis System and Power Supply-EPS601 (GE Healthcare), following a protocol as reported earlier (Jessie, Hashim & Rahim, 2008). The 2DE gels were then developed by silver staining (Heukeshoven & Dernick, 1988). All samples (at each time point) were examined independently in triplicate. A modified silver staining protocol was performed for visualization of proteins well-suited for MALDI-ToF/ToF mass spectrometry (MS) investigation (Yan et al., 2000).

### Image analysis

2DE gels (silver stained) were scanned using Imaging Densitometer GS690 (Bio-Rad Laboratories, Hercules, CA, USA). Expression level of proteins was calculated in terms of percentage of volume contribution using the Image Master 2D Platinum software, version 7.0 (GE Healthcare Biosciences, Uppsala, Sweden) by selecting the particular spot of the 2DE, matching it with the same spot of another replicate (of the same time point) for which a matchset has been already created using the software. Cut-off parameters for this analysis were: Smooth—2; Saliency—1; Min area—5 (Jayapalan et al., 2012; Jayapalan et al., 2013).

### Trypsin digestion and mass spectrometry

Identification of protein spots of interest was performed as described previously (Jayapalan et al., 2012; Jayapalan et al., 2013). Further, spots were carefully excised from 2DE gels and destained with potassium ferricyanide (15 mM) and sodium thiosulfate (50 mM) for 20 min at about 20 °C. The proteins were reduced with 10 mM DTT (10 mM) and ammonium bicarbonate (100 mM) for 25 min, and alkylated with 55 mM iodoacetamide (55 mM) for 15 min, at 60 °C and in the dark, respectively. This was followed by subsequent washings with 50 and 100% acetonitrile (ACN, 50 and 100%) in 100 mM ammonium bicarbonate (100 mM), and dehydration of the gel plugs using vacuum centrifugation. The spots were digested with trypsin (6 ng/µl in ammonium bicarbonate solution (50 mM)) at 37 °C, for about 12 h. Peptides were extracted from the gels using ACN (50 and 100 %) subsequently. Extracted peptides were lyophilized, treated with formic acid (0.1%) and desalted using ZipTip columns containing C^18^ reversed phase media (Millipore, Madison, USA). The sample peptide was mixed with *α*-cyano-4-hydroxycinamic acid (5 mg/ml) at a ratio of 1:1, and 0.7 µl of the mixture was immediately spotted onto an OptiToF 384-well insert and analyzed using 5,800 MALDI ToF/ToF analyser (ABSciex, Toronto, Canada).

### Identification of proteins

The proteins in the spots/clusters were identified using the MASCOT search engine (Jayapalan et al., 2012; Jayapalan et al., 2013). The MS data acquired was searched against *Drosophila melanogaster* in the Swiss-Prot database (last update: 21 April 2015, 3,067 sequences) in accordance with the following selection parameters: enzyme-trypsin, missed cleavage—1, variable modification—2; (i) carbamidomethylation of cysteine and (ii) oxidation of methionine, MS precursor ion mass tolerance—100 ppm, MS/MS fragment ion tolerance −0.2 Da and inclusion of monoisotopic masses only.

### Statistical analysis

Percentage of volume contribution was expressed as mean ± SD. The Statistical Package for Social Sciences (SPSS) version 22.0 (IBM Corporation, New York, NY, USA) was used to analyze the data. The test of homogeneity was employed to evaluate the sample distribution of the dataset. In the study, maximum numbers of protein spots were recorded at 04:00 and 20:00. Hence, the percentages of volume contribution of spots/clusters of these time points were used for comparison with other time points. The Student’s *t*-test was subsequently used to compare means of percentage of volume contribution of the spots (04:00 and 20:00) with other time points (individually) of all datasets that showed normal distribution. A *p* value of <0.01 was deemed significant.

## Results

**Figure 1:**
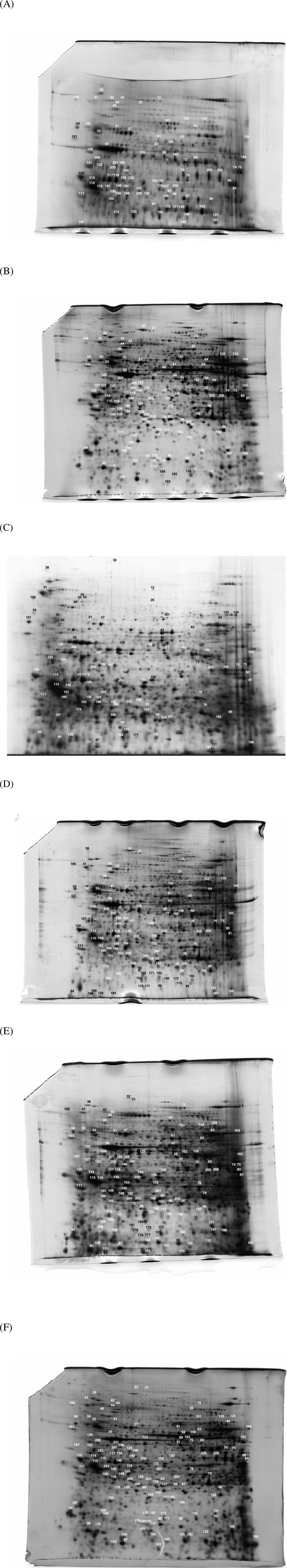
Representative 2DE photographs of proteome profile of Drosophila melanogaster at 4-h intervals over a period of 24-h. The protein spots/cluters are labeled in black (or) white for easy visualization. (A) 00:00, (B) 04:00, (C) 08:00, (D) 12:00, (E) 16:00 and (F) 20:00. At each time point, 2DE was performed in triplicate. Expression levels of the spots/clusters are designated as percentage of volume contribution using the Image Master 2D Platinum software, version 7.0.

Figures 1A–1F demonstrate the representative 2DE profiles of *D. melanogaster* at 00:00, 04:00, 08:00, 12:00, 16:00 and 20:00. Marked variations in intensity of the protein spots/ clusters were apparent over the 24 h period. A total of 124 protein spots/clusters, which were classified into 11 groups based on known or predictive functions, were identified by MS/MS analysis and database query (Table 1). In this analysis, matched peptides for 2 or more reflect better confidence of the results. A few protein spots, which were not resolved at all time points, were excluded from the analysis. Multiple hits for single protein spots (e.g., spot/cluster Nos. 57 and 200, 104 and 135, 86 and 175 and 120 and 204) were also observed.

**Figure 2:**
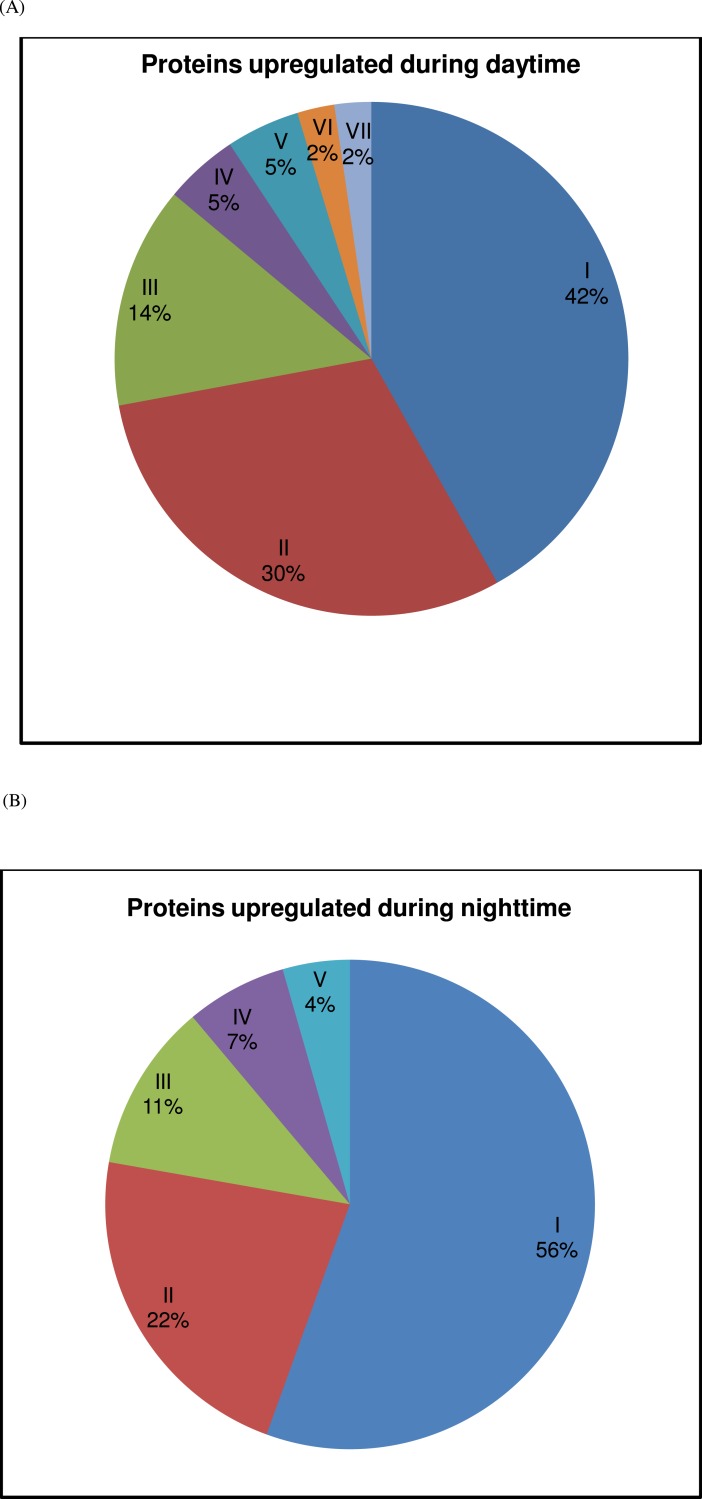
Contribution of protein groups over the 24-h period. (A) The contribution of upregulated proteins of each group (I—metabolism, II—muscle activity, III—ion-channel/cellular transport, IV—protein synthesis/folding/degradation, V—redox homeostasis, VI—development and VII—transcription) during daytime (representing 08:00, 12:00 and 16:00) is represented. (B) The contribution of upregulated proteins of each group (I—metabolism, II—muscle activity, III—heat shock proteins, IV—protein synthesis/folding/degradation and V—apoptosis) during nighttime (representing 20:00, 00:00 and 04:00) is represented. See Table 2 for further details.

**Figure 3:**
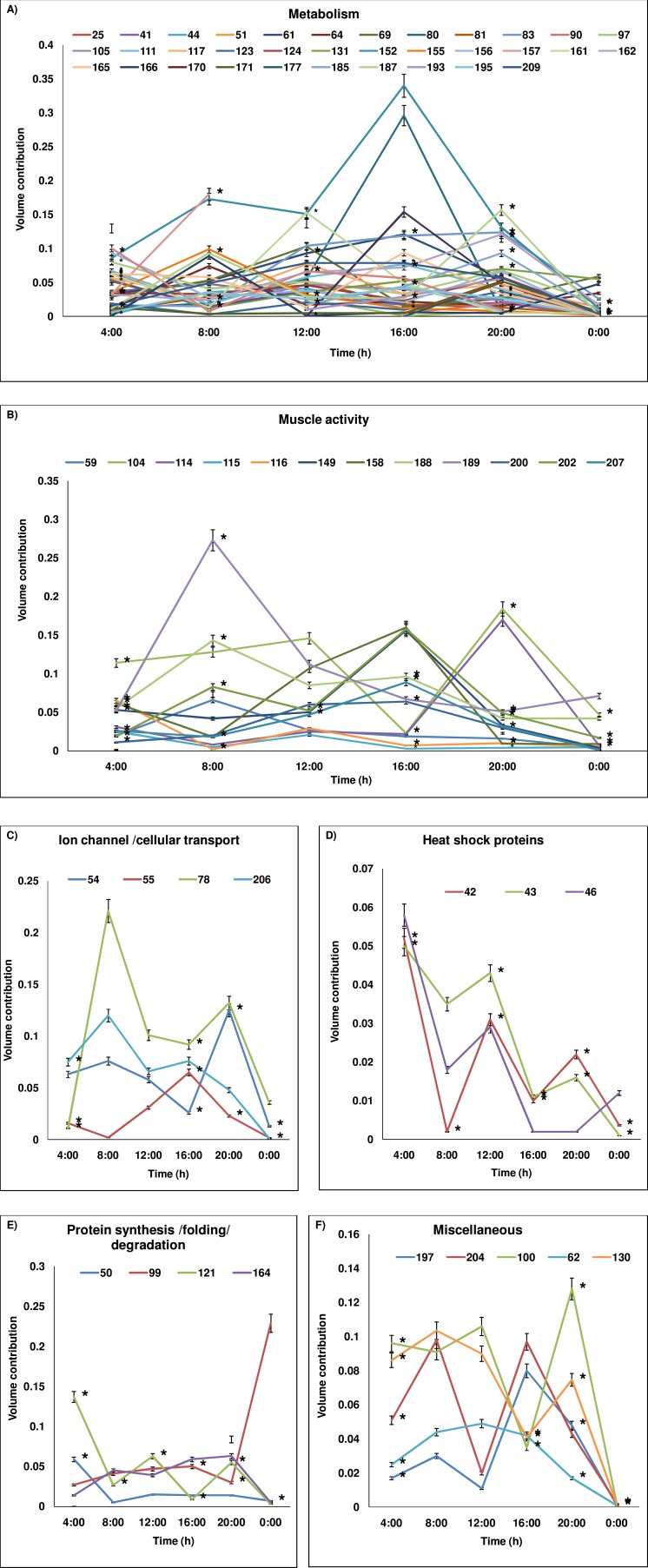
Temporal variation in expression level of proteins. Protein level variations (as percentage of volume contribution) of the groups across 24-h period are shown. (A) metabolism, (B) muscle activity, (C) ion-channel/cellular transport, (D) heat shock proteins, (E) protein synthesis/folding/degradation, (F) miscellaneous. The proteins which show expression at all time points are represented and protein spot/cluster number is given in the figure. The mean ± SD values of percentage of volume contribution are plotted. At some time points, the SD values are nearly 0 and hence the values may not be visible in the reduced scale. The time points at which the expression is significantly different are marked with *. In (A) as numerous temporal variations are plotted, the SD values and * marks are plotted only for certain proteins for easy visualization. The SD values and significant variations of all proteins (A–F) are given in Table 2.

When image analysis of the 2DE gels was performed, expression of 88 proteins was found to demonstrate significant variation over the 24 h period (Table 2). Whilst 45 appeared upregulated during the nighttime, 43 were overexpressed in the daytime (Table 2 and Fig. 2). The complexity of temporal regulation of *D. melanogaster* proteome was further reflected from functional annotations of the differently expressed proteins, with those that were upregulated during the nighttime being restricted to the heat shock proteins and proteins involved in metabolism, muscle activity, protein synthesis/folding/degradation and apoptosis, whilst those that were overexpressed in the daytime were apparently involved in metabolism, muscle activity, ion-channel/cellular transport, protein synthesis/folding/degradation, redox homeostasis, development and transcription. In addition, proteins which showed significant variation in percentage of volume contribution in at least 2 time points were apparently from six different functional groups (i.e., metabolism, muscle activity, ion-channel/cellular transport, heat shock proteins, protein synthesis/folding/degradation and miscellaneous (redox homeostasis, apoptosis, development and transcription); (Table 2 and Figs. 3A–3I). The percentage of volume contribution of all 124 protein spots/clusters are shown in the Table S1.

**Table 1:** Identification of *Drosophila melanogaster* proteins that were differentially expressed by mass spectrometry. The MS data acquired was searched in Swiss-Prot database and the proteins were identified using the MASCOT search engine. The proteins are categorized into several groups based on known or predictive functions.

S. No.	Spot/ Cluster ID/No.	Protein identification	Primary accession number	Theoretical mass (Da)	Calculated pI	Peptide score	No. of peptides matched	Sequence coverage (%)
		**Metabolism**						
1	12	6-phosphofructokinase	P52034	86593	6.41	43	3	5
2	25	Succinate dehydrogenase	Q94523	72297	6.65	149	10	21
3	32	N-glycanase	Q28YQ7	73443	8.15	26	1	3
4	41	Maltase H	P07190	66344	4.75	82	5	10
5	44	Vacuolar ATP synthase catalytic subunit A	Q27331	68259	5.23	148	13	22
6	51	Vacuolar ATP synthase subunit B	P31409	54515	5.25	131	10	27
7	61	Enolase	P15007	54276	8.68	415	11	32
8	63	Phosphoglycerate kinase	Q01604	43834	7.01	223	6	18
9	64	ATP synthase subunit *α*	ACP35381	59384	9.09	332	5	15
10	68	Arginine kinase	P48610	39841	6.04	363	7	26
11	69	Fructose bisphosphate aldolase	P07764	39023	6.97	525	10	34
12	71	Arginine kinase	P48610	39841	6.04	291	10	38
13	72	Glyceraldehyde-3-phosphate dehydrogenase	P07486	35328	8.26	46	2	8
14	74	Glyceraldehyde-3-phosphate dehydrogenase	P07486	35328	8.26	332	5	25
15	75	Glyceraldehyde-3-phosphate dehydrogenase 1	P07486	35328	6.44	306	3	15
16	79	Alcohol dehydrogenase	P00334	27744	7.64	358	6	43
17	80	Stellate protein CG33247	Q7KV23	19396	7.64	28	1	5
18	81	Stellate protein CG33247	Q7KV23	19396	7.64	28	1	5
19	83	Triosephosphate isomerase	Q7JNS1	26609	6	178	7	43
20	90	ATP synthase subunit *β*	Q05285	54074	5.14	67	5	8
21	97	ATP synthase D chain	Q24251	20188	6.1	81	5	33
22	98	Tyrosine kinase 2	Q9V3D5	79559	9.13	23	1	1
23	105	ATP synthase subunit *β*	Q05825	54074	5.14	178	7	16
24	111	Protein l(2)37Cc	P18432	23700	4.67	56	1	6
25	117/118	ATP synthase subunit *β*	Q05825	54074	5.14	573	11	28
26	123	ATP synthase subunit *β*	Q05825	54074	5.14	61	6	17
27	124	Inorganic pyrophosphatase	O77460	37915	6.52	99	3	12
28	126	ATP synthase subunit *β*	Q05825	54074	5.14	48	2	3
29	131	Fructose bisphosphate aldolase	P07764	39023	6.97	92	5	21
30	132	Alcohol dehydrogenase	P00334	27744	7.74	198	5	28
31	133	Pyruvate kinase	O62619	57404	7.13	59	3	7
32	140	Phosphoglycerate kinase	Q01604	43834	7.01	195	7	15
33	152	Phosphoglycerate kinase	Q01604	43834	7.01	217	5	15
34	155	Glycerol-3-phosphate dehydrogenase	P13706	39659	6.17	96	7	26
35	156	Isocitrate dehydrogenase	Q9VWH4	40818	6.96	63	7	21
36	157	Glyceraldehyde-3-phosphate dehydrogenase	P07486	35328	8.26	106	2	8
37	159	ATP synthase subunit *α*	P35381	59384	9.09	380	10	20
38	161	Thioredoxin reductase I	P91938	64282	8.11	79	7	12
39	162	Fructose bisphosphate aldolase	P07764	39023	6.97	55	3	16
40	163	Alcohol dehydrogenase	P00334	27744	7.74	105	3	18
41	165	ATP synthase subunit *α*	P35381	59384	9.09	196	5	14
42	166	Maltase	P07190	66344	4.75	92	7	15
43	170	Pyruvate kinase	O62619	57404	7.13	35	2	4
44	171	Glycerol-3-phosphate dehydrogenase	Q27556	38298	6.33	27	6	20
45	173	ATP synthase subunit *β*	Q05825	54074	5.14	263	7	14
46	177	Fructose bisphosphate aldolase	P07764	39023	6.97	22	7	22
47	178	ATP synthase subunit *α*	P35381	59384	9.09	77	3	6
48	183	Alcohol dehydrogenase	P00334	27744	7.74	22	1	7
49	185	ATP synthase subunit *β*	Q05825	54074	5.14	32	1	2
50	186	Fructose bisphosphate aldolase	P07764	39023	6.97	176	6	16
51	187	Fructose bisphosphate aldolase	P07764	39023	6.97	107	4	13
52	192	Glyceraldehyde-3-phosphate dehydrogenase 1	P07486	35328	8.26	40	3	12
53	193	Cytochrome P450	Q9VGB5	55583	9.39	27	2	2
54	195	Fructose bisphosphate aldolase	P07764	39023	6.97	62	4	15
55	196	Fructose bisphosphate aldolase	P07764	39023	6.97	58	4	17
56	199	Inorganic pyrophosphatase	O77460	37915	6.52	93	5	19
57	209	Arginine kinase	P48610	39841	6.04	50	1	2
		**Muscle activity**						
58	57	Actin-87E	P10981	41775	5.3	132	6	20
59	59	Actin-88F	P83967	41673	5.29	421	7	26
60	94	Myosin light chain alkali	P06742	17513	4.29	89	2	16
61	102	Actin-57B	P53501	41808	5.23	22	5	17
62	104	Actin-88F	P83967	41673	5.29	413	5	22
63	106	Actin-5C	P10987	41795	5.3	27	2	6
64	114	Myosin regulatory light chain-2	P10987	41795	5.3	215	6	20
65	115	Actin-5C	P02574	41760	5.3	194	4	14
66	116	Actin-79B	Q540X7	41760	5.3	194	4	14
67	125	Actin-5C	P10987	41795	5.3	30	2	6
68	135	Actin-88F	P83967	41673	5.29	316	6	20
69	136	Actin-5C	P10987	41795	5.3	25	2	5
70	149	Actin-5C	P10987	41795	5.3	182	3	11
71	151	Synapse associated protein	Q960T2	56946	4.45	46	1	1
72	153	Actin-5C	P10987	41795	5.3	27	1	2
73	154	Tubulin *α*-1 chain	P06603	49876	5	173	5	14
74	158	ADP-ribosylation factor-8	Q9VHV5	21240	6.74	28	1	4
75	168	Actin-79B	P02574	41760	5.3	190	8	23
76	169	Actin-88F	P83967	41673	5.29	43	2	9
77	172	Tubulin *β*-1 chain	Q24560	50115	4.76	30	2	3
78	174	Actin-57B	P53501	41808	5.23	117	3	10
79	182	Actin-87E	P10981	41775	5.3	46	2	7
80	184	Actin-57B	P53501	41808	5.23	136	5	15
81	188	Actin-57B	P53501	41808	5.23	103	5	17
82	189	Actin-5C	P10987	41795	5.3	108	4	14
83	191	Actin-42A	P02572	41797	5.3	41	2	7
84	194	Tubulin *α*-3 chain	P06605	49859	5	78	1	3
85	198	Actin-79B	P83967	41760	5.29	118	5	20
86	200	Actin-87E	P10981	41775	5.3	194	5	20
87	201	Actin-57B	P53501	41808	5.23	112	3	10
88	202	Paramyosin	P35415	102277	5.47	36	5	7
89	203	Actin-42A	P02572	41797	5.3	83	2	6
90	208	Actin-5C	P10987	41795	5.3	45	3	9
		**Heat shock proteins**						
91	31	Heat shock 82 kDa protein	P02828	81814	4.91	23	4	9
92	42	Heat shock 70 kDa protein	P29844	72216	5.22	84	6	12
93	43	Heat shock 70 kDa protein	P11147	71087	5.36	188	8	16
94	46	Heat shock 60 kDa protein	O02649	60771	5.38	26	1	1
95	180	Heat shock factor protein	P22813	76886	4.87	27	1	3
		**Ion-channel/cellular transport**						
96	54	Calreticulin	P29413	46779	4.4	89	2	5
97	55	Tubulin *β*-1 chain	Q24560	50115	4.76	149	9	19
98	78	Voltage-dependent anion-selective channel (Porin)	Q94920	30531	7.74	387	7	36
99	96	Calcium-transporting ATPase	P22700	111630	5.28	27	1	1
100	160	ADP/ATP translocase	Q26365	34193	9.82	247	6	14
101	181	Voltage-dependent anion-selective channel (Porin)	Q94920	30531	6.44	195	4	20
102	205	Voltage-dependent anion-selective channel (Porin)	Q94920	30531	6.44	22	1	3
103	206	Voltage-dependent anion-selective channel (Porin)	Q94920	30531	6.44	27	1	3
104	207	Transient receptor potential locus C protein	P36951	29075	6.07	24	2	11
		**Redox homeostasis**						
105	85	Capon-like protein	Q8SXX4	77233	8.98	22	1	1
106	86	Superoxide dismutase [Cu–Zn]	P61851	15689	5.67	162	8	62
107	120	Glutathione-S-transferase	P41043	27596	4.57	224	5	19
108	134	Catalase	ACP17336	57113	8.39	54	4	9
109	175	Superoxide dismutase [Cu–Zn]	P61851	15689	5.67	56	3	24
110	197	Glutathione-S-transferase	P41043	27596	4.57	74	3	9
111	204	Glutathione-S-transferase	P20432	23851	6.75	61	1	4
		**Protein synthesis/folding/degradation**						
112	50	Protein disulfide isomerase	P54399	55746	4.72	65	2	5
113	99	Furin-like protease	P30430	120917	6.25	22	1	1
114	121	40S ribosomal protein SA	P38979	30209	4.76	50	2	9
115	164	Elongation factor 1*α* (EF-1*α*)	P08736	59384	9.09	52	4	9
116	167	40S ribosomal protein SA	P38979	30209	4.76	38	2	9
117	179	40S ribosomal protein S12	P80455	15159	5.93	32	1	10
		**Miscellaneous**						
118	100	Thioredoxin peroxidase (apoptosis)	Q9V3P0	21724	5.52	122	4	26
119	176	Caspase-8 precursor (apoptosis)	Q29IM7	57539	6.31	22	1	3
120	62	Vitellogenin-2 precursor (development)	ACP02844	49630	7.74	56	6	12
121	87	Protein stand still (development)	P92189	35753	9.28	26	1	2
122	190	DNA polymerase *α*-catalytic subunit (replication)	P26019	169796	8.28	33	1	0
123	130	RNA helicase (transcription)	Q6J5K9	144797	5.67	30	1	0
124	38	Mitosis initiation protein fs(1)Ya (cell division)	P25028	77677	9.54	21	1	2

Among the protein spots which showed significant variations over the 24 h period are various enzymes involved in metabolism ((6-phosphofructokinase, succinate dehydrogenase, N-glycanase, maltase H, vacuolar ATP synthase catalytic subunits A and B, enolase, ATP synthase subunits (*α*, *β* and D chain), fructose bisphosphate aldolase, arginine kinase, stellate protein CG33247 (protein kinase regulator), triosephosphate isomerase, protein l(2)37Cc (DOPA decarboxylase), inorganic pyrophosphatase, pyruvate kinase, phosphoglycerate kinase, glycerol-3-phosphate dehydrogenase, isocitrate dehydrogenase, thioredoxin reductase, alcohol dehydrogenase and cytochrome P450)). Others which showed significant variation of expression at different time points include: (i) proteins involved in muscular activities (various types of actin (88F, 57B, 5C, 79B, 87E and 42A), myosin regulatory light chain-2, synapse associated protein, ADP-ribosylation factor-8, tubulin *α*-3 chain and paramyosin), (ii) ion-channel/proteins involved in transport processes (calreticulin, tubulin *β*-1 chain, porin, ADP/ATP translocase and transient receptor potential locus C protein), (iii) different types of heat shock proteins (82 kDa, 70 kDa, 60 kDa and heat shock factor protein), (iv) proteins associated with synthesis/folding/degradation (protein disulfide isomerase, furin-like protease, 40 S ribosomal protein SA and S12 and elongation fator 1*α* (EF-1*α*), (v) redox homeostasis protein (glutathione-S-transferase) (vi) apoptosis proteins (caspase-8 precursor and thioredoxin peroxidase (also as antioxidant)), (vii) proteins involved in development (vitellogenin-2 precursor) and transcription (RNA helicase) (Table 2 and Figs. 3B–3I).

**Table 2:** Expression levels of proteins as percentage of volume contribution by image analysis (ImageMaster 2D Platinum software, version 7.0). The proteins in the groups (metabolism, muscle activity, ion-channel/cellular transport, heat shock, protein synthesis/folding/degradation, and miscellaneous (redox homeostasis, apoptosis, development and transcription)) which show significant variation in at least 2 time points are included in the table. At each time point, 2-DE was performed in triplicate. For other details see Table S1.

S. No.	Spot/ Cluster ID/No.	Protein name	Significance level of % volume^a^					
			04:00	08:00	12:00	16:00	20:00	00:00
		**Metabolism**						
1	12	6-phosphofructokinase	0.0168 ± 0.080		0.013871 ± 0.0093 *p* = 0.00017^c^		0.00355 ± 0.00336 *p* = 0.00026^d^	
2	25	Succinate dehydrogenase	0.05666 ± 0.03670			0.01156 ± 0.01067 *p* = 6.8929E–05^b^	0.02717 ± 0.01942 *p* = 0.00980^d^	
3	32	N-glycanase	0.04920 ± 0.02726		0.02594 ± 0.01233 *p* = 0.00870^b^	0.00113 ± 0.00013 *p* = 2.5021E–05^c^	0.00715 ± 0.00467 *p* = 6.7294E–07^d^	
4	41	Maltase H	0.03721 ± 0.02519	0.04786 ± 0.06067	0.03036 ± 0.01416	0.01673 ± 0.01416	0.0195 ± 0.01690	0.000583 ± 0.00065 *p* = 0.00036^b^*p* = 0.00233^c^
5	44	Vacuolar ATP synthase catalytic subunit A	0.00312 ± 0.02442	0.03587 ± 0.06408	0.04102 ± 0.03517	0.01178 ± 0.01333	0.02862 ± 0.01673	0.0080 ± 0.0015 *p* = 0.00771^b^*p* = 0.00177^c^
6	51	Vacuolar ATP synthase catalytic subunit B	0.08049 ± 0.05789		0.06707 ± 0.03213 *p* = 0.00198^c^		0.04721 ± 0.01989	0.01295 ± 0.00224 *p* = 7.1834E–05^b^*p* = 0.00198^c^
7	61	Enolase	0.03289 ± 0.00521	0.05179 ± 0.07539	0.09340 ± 0.08054 *p* = 0.00139^b^	0.12059 ± 0.11632 *p* = 0.00239^b^	0.0561 ± 0.07604	0.00145 ± 0.00177
8	64	ATP synthase subunit *α*	0.01221 ± 0.01438	0.07412 ± 0.00754 *p* = 7.3863E–08^b^*p* = 5.7179E–06^c^	0.01719 ± 0.0472	0.02236 ± 0.04303	0.01559 ± 0.00272	0.0345 ± 0.02371
9	69	Fructose bisphosphate aldolase	0.02904 ± 0.00464	0.05306 ± 0.03179	0.10312 ± 0.00128 *p* = 0.00186^b^*p* = 0.00093^c^	0.0154 ± 0.0300	0.02551 ± 0.00406	0.05898 ± 0.02373
10	71	Arginine kinase	0.12971 ± 0.07577		0.13765 ± 0.02036 *p* = 0.00838^c^		0.04893 ± 0.04735	0.00027 ± 0.00018 *p* = 0.0010^b^
11	80	Stellate protein CG33247	0.01139 ± 0.00267	0.0197 ± 0.0438	0.05522 ± 0.01000 *p* = 0.00483^b^	0.0296 ± 0.0644	0.00743 ± 0.01502	0.00266 ± 0.00303
12	81	Stellate protein CG33247	0.00784 ± 0.00121	0.02921 ± 0.0239	0.05163 ± 0.00106 *p* = 0.00507^b^	0.01766 ± 0.0354	0.0129 ± 0.02987	0.00360 ± 0.00349
13	83	Triosephosphate isomerase	0.01722 ± 0.07347	0.01545 ± 0.06742	0.10425 ± 0.08459	0.11867 ± 0.08459	0.12435 ± 0.02904	0.02646 ± 0.01363 *p* = 0.00061^c^
14	90	ATP synthase subunit *β*	0.0121 ± 0.00155			0.00539 ± 0.00112 *p* = 0.00247^b^	0.019 ± 0.03214 *p* = 0.00024^d^	
15	97	ATP synthase D chain	0.08024 ± 0.01850	0.04083 ± 0.0364	0.05072 ± 0.04827	0.03286 ± 0.02797	0.06776 ± 0.06139	0.01847 ± 0.00439 *p* = 0.00263^b^
16	105	ATP synthase subunit *β*	0.06354 ± 0.05091	0.01256 ± 0.03672	0.04666 ± 0.05516	0.0397 ± 0.02289	0.02342 ± 0.03046	0.00179 ± 0.00281 *p* = 0.00151^b^
17	111	Protein l(2)37Cc	0.06736 ± 0.04438	0.018423 ± 0.00270 *p* = 0.00809^b^	0.05768 ± 0.02585	0.07649 ± 0.10300	0.0401 ± 0.03811	0.00559 ± 0.00881 *p* = 0.00022^b^*p* = 0.00787^c^
18	117	ATP synthase subunit *β*	0.05724 ± 0.00662	0.02367 ± 0.0637	0.07744 ± 0.09473	0.0326 ± 0.06556	0.0476 ± 0.0820	0.00466 ± 0.00134 *p* = 0.00096^b^
19	123	ATP synthase subunit *β*	0.01906 ± 0.01613	0.00331 ± 0.00433	0.02062 ± 0.0099	0.01013 ± 0.0012	0.03737 ± 0.02032	0.00281 ± 0.00336 *p* = 0.00080^c^
20	124	Inorganic pyrophosphatase	0.03386 ± 0.02401	0.0325 ± 0.01715	0.04636 ± 0.02574	0.02309 ± 0.02131	0.05833 ± 0.00977	0.00568 ± 0.00112 *p* = 0.00721^b^*p* = 1.5608E–06^c^
21	131	Fructose bisphosphate aldolase	0.05415 ± 0.05416	0.01170 ± 0.02879 *p* = 0.00780^c^	0.03636 ± 0.01339	0.0522 ± 0.07434	0.0694 ± 0.00939	0.054637 ± 0.00665
22	133	Pyruvate kinase	0.05199 ± 0.03744	0.0145 ± 0.01690	0.0377 ± 0.0492	0.01838 ± 0.01070 *p* = 0.00287^b^		
23	152	Phosphoglycerate kinase	0.08607 ± 0.05137	0.17323 ± 0.03216	0.14976 ± 0.02226	0.34022 ± 0.07880 *p* = 0.00945^b^	0.13099 ± 0.00249	0.01186 ± 0.00197 *p* = 0.00016^c^
24	155	Glycerol-3-phosphate dehydrogenase	0.03523 ± 0.01054	0.0993 ± 0.05331	0.03185 ± 0.01802	0.01244 ± 0.00796	0.00780 ± 0.00056 *p* = 0.00706^d^	0.00171 ± 0.00257 *p* = 1.6430E–05^b^*p* = 0.00440^c^
25	156	Isocitrate dehydrogenase	0.09404 ± 0.06044	0.02818 ± 0.00736	0.03816 ± 0.0096	0.02102 ± 0.06067	0.06589 ± 0.10657	0.00184 ± 0.00023 *p* = 5.3619E–05^b^
26	157	Glyceraldehyde-3-phosphate dehydrogenase	0.03387 ± 0.00190	0.17997 ± 0.03068 *p* = 0.00119^b^			0.11537 ± 0.00203 *p* = 9.0253E–07^d^	
27	161	Thioredoxin reductase I	0.06182 ± 0.03565	0.01353 ± 0.01329	0.15359 ± 0.05446 *p* = 0.00536^c^	0.04874 ± 0.01566	0.01517 ± 0.00499	0.00059 ± 0.00048 *p* = 0.00265^b^*p* = 0.00012^c^
28	162	Fructose bisphosphate aldolase	0.03210 ± 0.01170	0.02430 ± 0.01086 *p* = 0.00021^c^	0.06483 ± 0.01934 *p* = 0.00315^b^*p* = 0.00938^c^	0.07216 ± 0.02846 *p* = 0.00248^b^	0.12061 ± 0.00710 *p* = 4.1997E–08^d^	0.01689 ± 0.00260 *p* = 0.00263^c^
29	163	Alcohol dehydrogenase				0.04532 ± 0.01706 *p* = 0.00964^c^	0.00838 ± 0.00187	
30	165	ATP synthase subunit *α*	0.06259 ± 0.02558	0.05752 ± 0.04504	0.01409 ± 0.01	0.09364 ± 0.01434	0.0328 ± 0.00413	0.00082 ± 0.00054 *p* = 0.00123^b^
31	166	Maltase H	0.00333 ± 0.00325	0.08944 ± 0.09212	0.00040 ± 0.00019 *p* = 0.00216^c^	0.00617 ± 0.00298	0.00480 ± 0.00155	0.04754 ± 0.03454
32	170	Pyruvate kinase	0.00480 ± 0.00251				0.02907 ± 0.00478 *p* = 0.00839^d^	
33	171	Glycerol-3-phosphate dehydrogenase	0.01344 ± 0.00408	0.00372 ± 0.00255 *p* = 0.00016^c^	0.00526 ± 0.00287 *p* = 0.00020^c^	0.00482 ± 0.00032 *p* = 0.00736^b^	0.05168 ± 0.00546 *p* = 0.00063^d^	0.00688 ± 0.00578 *p* = 0.00063^b^
34	173	ATP synthase subunit *β*			0.00046 ± 0.00031 *p* = 0.00025^c^	0.15433 ± 0.02157 *p* = 0.00126^c^	0.04854 ± 0.00678	
35	177	Fructose bisphosphate aldolase	0.00406 ± 0.00075			0.00050 ± 0.00027 *p* = 0.00153^b^*p* = 0.00030^c^	0.04982 ± 0.00730 *p* = 0.00041^d^	
36	178	ATP synthase subunit *α*	0.00288 ± 0.00394			0.00459 ± 0.00615 *p* = 0.00025^c^	0.05003 ± 0.00671 *p* = 3.2629E–06^d^	
37	185	ATP synthase subunit *β*	0.00871 ± 0.00543		0.01777 ± 0.00956 *p* = 1.7782E–06^c^	0.04401 ± 0.01014 *p* = 0.00020^b^*p* = 0.00029^c^	0.09301 ± 0.00422 *p* = 5.0701E–09^d^	0.00877 ± 0.00187 *p* = 1.0732E–05^c^
38	186	Fructose bisphosphate aldolase	0.10034 ± 0.05736	0.00754 ± 0.01134 *p* = 2.2442E–07^b^	0.06666 ± 0.02744 *p* = 0.00087^c^	0.05637 ± 0.02768 *p* = 0.00536^c^	0.00989 ± 0.02621 *p* = 5.4053E–06^d^	
39	187	Fructose bisphosphate aldolase	0.01225 ± 0.01942	0.09305 ± 0.03952 *p* = 0.00727^b^	0.02897 ± 0.01348 *p* = 0.00241^c^	0.02634 ± 0.04315 *p* = 0.00226^c^	0.15707 ± 0.06963 *p* = 0.00277^d^	0.00230 ± 0.00212 *p* = 2.0640E–05^c^
40	193	Cytochrome P450	0.00662 ± 0.00730		0.00985 ± 0.00105 *p* = 0.00174^b^*p* = 0.00028^c^	0.02990 ± 0.00493 *p* = 0.00012^c^	0.05572 ± 0.00066 *p* = 1.0684E–06^d^	
41	195	Fructose bisphosphate aldolase	0.00581 ± 0.00388	0.03529 ± 0.05554	0.02590 ± 0.00795 *p* = 0.00391^b^	0.04218 ± 0.00960 *p* = 0.00090^b^	0.02665 ± 0.02258	0.00053 ± 0.00047 *p* = 0.00139^b^*p* = 0.00307^c^
42	199	Inorganic pyrophosphatase	0.02621 ± 0.01431				0.04274 ± 0.01619	0.00144 ± 0.00105 *p* = 1.0825E–06^b^*p* = 5.2773E–09^c^
43	209	Arginine kinase	0.01591 ± 0.00931	0.04940 ± 0.01749 *p* = 0.00610^b^	0.07943 ± 0.01468 *p* = 2.8958E–05^b^	0.079384 ± 0.03733 *p* = 0.00411^b^	0.05711 ± 0.00992 *p* = 0.00047^d^	0.00402 ± 0.00257 *p* = 0.00013^c^
		**Muscle activity**						
44	59	Actin-88F	0.02046 ± 0.00313	0.06569 ± 0.00868 *p* = 5.8021E–05^b^	0.0269 ± 0.07303	0.01924 ± 0.04082	0.01591 ± 0.02909	0.0039 ± 0.00216 *p* = 1.1928E–06^b^*p* = 1.1046E–05^c^
45	102	Actin-57B	0.07233 ± 0.01558		0.02407 ± 0.01990 *p* = 0.00101^b^		0.05179 ± 0.02690	0.00096 ± 0.00085 *p* = 5.0262E–07^b^*p* = 0.00038^c^
46	104	Actin-88F	0.11418 ± 0.01078	0.12801 ± 0.01392	0.14641 ± 0.11335	0.0227 ± 0.00535 *p* = 0.00738^b^*p* = 0.00032^c^	0.18435 ± 0.17218	0.04722 ± 0.03402
47	114	Myosin regulatory light chain-2	0.03078 ± 0.00338	0.00768 ± 0.02413	0.02481 ± 0.08161	0.02176 ± 0.05122	0.0174 ± 0.0255	0.00723 ± 0.00110 *p* = 0.00103^b^
48	115	Actin-5C	0.02452 ± 0.00371	0.00461 ± 0.00065 *p* = 0.00750^b^	0.02079 ± 0.0455	0.00308 ± 0.00072 *p* = 4.9214E–05^b^	0.00398 ± 0.00122 *p* = 0.00549^d^	0.00535 ± 0.00147 *p* = 0.00911^b^
49	116	Actin-79B	0.06538 ± 0.00565	0.00187 ± 0.00272 *p* = 7.981E–06^b^*p* = 0.00435^c^	0.02916 ± 0.08442	0.00724 ± 0.00169 *p* = 1.6044E–07^b^	0.01044 ± 0.00186 *p* = 2.6139E–06^d^	0.00615 ± 0.00132 *p* = 3.6987E–07^b^
50	149	Actin-5C	0.05339 ± 0.03502	0.04162 ± 0.0414	0.05021 ± 0.04663	0.15640 ± 0.19589	0.0328 ± 0.04848	0.00433 ± 0.00909 *p* = 0.00601^b^
51	151	Synapse associated protein			0.01538 ± 0.0055 *p* = 0.00118^c^	0.00974 ± 0.00762 *p* = 0.00132^c^	0.03087 ± 0.00508	
52	158	ADP-ribosylation factor-8	0.06229 ± 0.03123	0.01840 ± 0.00531 *p* = 0.00325^c^	0.10726 ± 0.01584	0.16042 ± 0.06361	0.09388 ± 0.02007	0.00920 ± 0.00118 *p* = 0.00746^b^*p* = 0.00025^c^
53	168	Actin-79B	0.00114 ± 0.00188		0.0201 ± 0.02050 *p* = 0.00921^b^		0.02964 ± 0.01304 *p* = 2.1258E–05^d^	
54	169	Actin-88F	0.00256 ± 0.00179				0.04922 ± 0.02446 *p* = 0.00191^d^	
55	174	Actin-57B	0.00193 ± 0.00344			0.04580 ± 0.01204 *p* = 0.00085^b^	0.03273 ± 0.00470 *p* = 0.00016^d^	0.00812 ± 0.00773 *p* = 0.00102^c^
56	182	Actin-87E	0.00355 ± 0.00395			0.05350 ± 0.03246 *p* = 0.00520^b^	0.07146 ± 0.01176 *p* = 9.2309E–07^d^	
57	184	Actin-57B	0.00062 ± 0.00095			0.12755 ± 0.00720 *p* = 1.3775E–07^b^*p* = 3.7756E–05^c^	0.0122 ± 0.02055	
58	188	Actin-57B	0.05530 ± 0.01465	0.14250 ± 0.00663 *p* = 0.00071^b^*p* = 4.5473E–05^c^	0.08487 ± 0.05086	0.09601 ± 0.01685 *p* = 0.00666^c^	0.04235 ± 0.00629	0.0421 ± 0.07126
59	189	Actin-5C	0.05170 ± 0.00922	0.27330 ± 0.07415 *p* = 0.00680^b^*p* = 0.00632^c^	0.11201 ± 0.05377	0.06672 ± 0.04820	0.0506 ± 0.0797	0.07088 ± 0.0666
60	194	Tubulin *α*-3 chain	0.00143 ± 0.00293		0.02587 ± 0.01795 *p* = 0.00082^b^*p* = 0.00204^c^	0.00259 ± 0.00353 *p* = 1.8164E–08^c^	0.07357 ± 0.00403 *p* = 8.0941E–09^d^	
61	198	Actin-79B	0.0135 ± 0.0029	0.05796 ± 0.00308 *p* = 5.6253E–05^b^		0.08917 ± 0.00759 *p* = 8.7657E–05^b^		
62	200	Actin-87E	0.0110 ± 0.01541	0.02004 ± 0.00506	0.05987 ± 0.00764 *p* = 0.00248^b^	0.06444 ± 0.05897	0.03010 ± 0.02593	0.00197 ± 0.00100 *p* = 0.00156^c^
63	201	Actin-57B					0.06669 ± 0.02571	0.00313 ± 0.00456 *p* = 4.3266E–05^c^
64	202	Paramyosin	0.01868 ± 0.00829	0.08292 ± 0.05254	0.05174 ± 0.00992 *p* = 0.00556^b^	0.15828 ± 0.04194 *p* = 0.00481^b^	0.04854 ± 0.0097	0.01727 ± 0.01595
65	203	Actin-42A		0.07337 ± 0.01193 *p* = 0.00736^c^			0.02297 ± 0.01262	
66	208	Actin-5C	0.03647 ± 0.01753	0.01795 ± 0.01412	0.04708 ± 0.01341	0.08915 ± 0.02907	0.03239 ± 0.00920	0.00092 ± 0.00019 *p* = 0.00407^c^
		**Ion-channel/cellular transport**						
67	54	Calreticulin	0.06318 ± 0.072311	0.07632 ± 0.01609	0.05745 ± 0.01702	0.02592 ± 0.01448 *p* = 0.00454^c^	0.12495 ± 0.02609	0.0131 ± 0.00333 *p* = 0.00012^c^
68	55	Tubulin *β*-1 chain	0.0158 ± 0.00237	0.00237 ± 0.0025	0.03131 ± 0.05206	0.06529 ± 0.00427 *p* = 0.00053^b^*p* = 0.00834^c^	0.0227 ± 0.02573	0.00163 ± 0.00122
69	78	Voltage-dependent anion-selective channel (Porin)	0.0109 ± 0.01073	0.2164 ± 0.0563	0.1006 ± 0.1049	0.0921 ± 0.01156 *p* = 0.00140^b^	0.13172 ± 0.11014	0.03589 ± 0.00776
70	160	ADP/ATP translocase	0.00215 ± 0.00150			0.0302 ± 0.02585 *p* = 0.00012^b^*p* = 9.6783E–07^c^	0.00391 ± 0.00784	
71	206	Voltage-dependent anion-selective channel (Porin)	0.07494 ± 0.06443	0.0116 ± 0.04136	0.06563 ± 0.00627	0.076146 ± 0.02511	0.04800 ± 0.10300	0.00101 ± 0.00116 *p* = 0.00581^b^
72	207	Transient receptor potential locus C protein				0.07783 ± 0.01334 *p* = 0.00394^c^	0.02824 ± 0.00534	0.00036 ± 0.00019 *p* = 4.0791E–08^c^
		**Heat shock proteins**						
73	31	Heat shock 82 kDa protein	0.02077 ± 0.01520		0.012483 ± 0.0060 *p* = 1.7087E–05^c^		0.00111 ± 0.00014 *p* = 0.00101^d^	
74	42	Heat shock 70 kDa protein	0.05238 ± 0.01099	0.00207 ± 0.00078 *p* = 0.00012^b^	0.0305 ± 0.00145 *p* = 0.00939^b^	0.00959 ± 0.00818 *p* = 5.2107E–05^b^	0.02227 ± 0.01378 *p* = 0.00187^d^	0.00374 ± 0.00089 *p* = 1.62E–08^b^*p* = 0.00028^c^
75	43	Heat shock 70 kDa protein	0.05023 ± 0.04775	0.03493 ± 0.00646	0.04306 ± 0.02875 *p* = 0.00157^c^	0.01095 ± 0.00139	0.01596 ± 0.01483 *p* = 0.00948^d^	0.00101 ± 0.00228 *p* = 8.70E–03^b^*p* = 8.95E–06^c^
76	46	Heat shock 60 kDa protein	0.05832 ± 0.03522	0.01751 ± 0.01643 *p* = 0.00400^b^	0.02924 ± 0.01869	0.00205 ± 0.02335 *p* = 0.00819^b^	0.00164 ± 0.00208 *p* = 0.00080^d^	0.01227 ± 0.00143 *p* = 0.00048^b^
77	180	Heat shock factor protein	0.01626 ± 0.00932				0.04055 ± 0.00227 *p* = 0.00755^d^	
		**Protein synthesis/folding/ degradation**						
78	50	Protein disulfide isomerase	0.05850 ± 0.00582	0.00525 ± 0.000696	0.01486 ± 0.00272	0.01374 ± 0.01066	0.01429 ± 0.02467	0.00699 ± 0.00148 *p* = 0.00237^b^
79	99	Furin-like protease	0.02738 ± 0.01222	0.04072 ± 0.01344	0.04672 ± 0.0098	0.05014 ± 0.00689 *p* = 0.00352^c^	0.03017 ± 0.00327	0.2291 ± 0.00774
80	121	40S ribosomal protein SA	0.13683 ± 0.01787	0.02732 ± 0.01356 *p* = 0.00107^b^*p* = 0.00908^c^	0.06246 ± 0.02463 *p* = 0.00708^b^	0.00885 ± 0.00078 *p* = 2.5433E–05^b^*p* = 2.0399E–08^c^	0.05494 ± 0.00225 *p* = 0.00023^d^	0.00305 ± 0.00589 *p* = 1.61E–06^b^*p* = 0.00022^c^
81	164	Elongation factor 1*α* (EF-1*α*)	0.01402 ± 0.01559	0.04487 ± 0.04267	0.03916 ± 0.03485	0.05944 ± 0.00664 *p* = 0.00147^b^	0.06306 ± 0.00781 *p* = 0.00098^d^	0.00452 ± 0.00442 *p* = 2.9002E–09^c^
82	179	40S ribosomal protein S12	0.00028 ± 0.00028				0.08388 ± 0.00936 *p* = 2.3206E–05^d^	
		**Miscellaneous**						
83	197	Glutathione-S-transferase (redox homeostasis)	0.01685 ± 0.00487	0.02996 ± 0.01557	0.01077 ± 0.00323	0.08026 ± 0.02642	0.04748 ± 0.03732	0.00108 ± 0.00099 *p* = 3.3424E–08^b^*p* = 0.00028^c^
84	204	Glutathione-S-transferase (redox homeostasis)	0.05135 ± 0.03322	0.09755 ± 0.05312	0.02023 ± 0.02641	0.09738 ± 0.04084	0.04298 ± 0.01096	0.00243 ± 0.00236 *p* = 0.00030^b^*p* = 1.1889E–07^c^
85	100	Thioredoxin peroxidase (apoptosis)	0.09598 ± 0.01127	0.09064 ± 0.10128	0.10623 ± 0.0569	0.03505 ± 0.01026 *p* = 0.00228^b^*p* = 8.085E–06^c^	0.12797 ± 0.00070 *p* = 0.00205^b^	0.00244 ± 0.00142 *p* = 1.2261E–06^b^*p* = 1.00E–07^c^
86	176	Caspase-8 precursor (apoptosis)	0.00092 ± 0.00013		0.00133 ± 0.00074 *p* = 4.1969E–06^c^	0.00473 ± 0.00655 *p* = 1.77E–06^c^	0.04464 ± 0.00337 *p* = 0.00012^d^	
87	62	Vitellogenin-2 precursor (development)	0.02446 ± 0.00269	0.0436 ± 0.0398	0.04901 ± 0.02959 *p* = 0.00238^b^*p* = 0.00149^c^	0.0417 ± 0.05467	0.01699 ± 0.00314	0.00053 ± 0.00009
88	130	RNA helicase (transcription)	0.08621 ± 0.03634	0.10347 ± 0.0988	0.09000 ± 0.01469	0.04088 ± 0.01000 *p* = 0.00092^c^	0.07469 ± 0.00027	0.00160 ± 0.00133 *p* = 0.00046^b^*p* = 0.00071^c^

**Notes.**

a Values are expressed in mean ± SD.

b Value compared with 04:00.

c Value compared with 20:00.

d Comparison of 04:00 and 20:00.

Protein spot IDs which showed different levels of expression at a minimum of any four time points were: 32, 42, 43, 46, 51, 59, 71, 100, 102, 115, 116, 121, 130, 152, 158, 161, 164, 171, 174, 176, 185, 187, 193, 194, 200 and 209. The enzymes involved in metabolism and muscular activities—fructose bisphosphate aldolase (187), arginine kinase (209), thioredoxin reductase I (161) and actin-87E (200) were upregulated during daytime and showed a lowest level of expression at 00:00. In addition, proteins/enzymes involved in various active cellular processes (i.e., (40S ribosomal protein SA (121), ATP synthase subunit *β* (185), heat shock protein 70 kDa (43), vacuolar ATP synthase subunit B (51), actin-88F (59), 57B (174) and 87E (200), thioredoxin peroxidase (100), RNA helicase (130), phosphoglycerate kinase (152), ADP-ribosylation factor-8 (158), and elongation factor-1*α* (164)) were apparently upregulated during the daytime but showed lowest level of expression at midnight (00:00). Conversely, proteins involved in apoptosis and toxin metabolism (caspase-8 precursor (176) and cytochrome P450 (193)) appeared upregulated at 20:00 compared to the daytime points (Figs. 3B–3I).

## Discussion

Living organisms perform their functions according to the light-dark cycle. Since, proteins and enzymes play vital roles in almost all the physiological functions of the body, a proper control of protein expression in a temporal manner is a crucial aspect of an organism. As complex interplay of multiple processes involved in the generation of overt rhythms of multiple biological functions, it is obvious that numerous proteins vary their expression in a temporal manner in the body of the fly. Studies on overall assessment of fly’s circadian proteome have confirmed the rhythmic nature of translation in *D. melanogaster* (Huang et al., 2013). However, it could be hypothesized that the circadian proteome is an outcome of regulatory stages at multiple steps of transcription and translation (RNA processing, posttranscriptional and posttranslational modifications). The conservation of circadian and clock controlled genes regulating similar pathways across various species, including *Drosophila* and mammals, is also well known (Akhtar et al., 2002). Since the majority of the regulatory mechanisms and signaling pathways are known to be conserved between *Drosophila* and humans (Rodriguez et al., 2013) the data generated in *Drosophila* may be applied to humans as well.

Our proteomics investigation demonstrated identification of 124 protein spots in the whole body of the fruit fly, *Drosophila melanogaster*. Eighty-eight of these proteins apparently showed temporal variation in expression. Analogous to our study, Rodriguez et al. (2013) had recognized more than 130 cycling transcriptional units in the heads of *D. melanogaster*, of which nearly one-third (44) cycled significantly. Among the 44, the peak times of mRNAs of cytochrome P450 (20:00 h) and glutathione-S-transferease (16:00) appeared synchronous with accumulation of their proteins that was observed in our study. However, the significant oscillations of several other proteins involved in metabolism, muscle activity, cellular transport, redox homeostasis, protein synthesis/folding/degradation, cell division and transcription that were also reported by Rodriguez et al. (2013) were not seen in our proteomics investigation. This may be partly due to the mRNA levels being investigated in heads of the fly by Rodriguez et al. (2013), whilst our analysis was performed on the whole body proteome.

The data of our study, when compared to other reports, demonstrated higher percentage of the fruit fly proteins under the clock control. For example, Reddy et al. (2006) had reported that only about 20% of soluble proteins in the proteome profile of whole mouse liver were under the circadian regulation. In addition, the proteome of suprachiasmatic nuclei (mammalaian circadian pacemaker) showed roughly 13% of soluble proteins demonstrated robust oscillations, with 53 of the protein spots in the 2DE proteome profile (Deery et al., 2009). In their study, more protein spots showed maximum expression during the day (65%) than night (35%). However, our study on the whole fly proteome showed slightly higher numbers of protein spots that were upregulated during nighttime than daytime.

The rhythmic protein abundance observed in the study may be caused by differences in the synthesis and/or half-life of proteins. In addition, it could also be attributed to (i) circadian transcriptional regulation by clock transcriptional factors and co-regulators which act on a wide array of circadian clock-controlled genes (ccgs) (Asher & Schibler, 2011), (ii) circadian hormonal signaling to various types of cells (Asher & Schibler, 2011; Lück et al., 2014) and (iii) rhythmically distinct feeding patterns (Vodala et al., 2012). Determination of the composition of proteins in the whole body of fruit fly is an essential step towards understanding the regulation of various proteins as an integrated system. In the whole body of *D. melanogaster* many genes showed coordinated circadian oscillations of expression but there were significant disconnections between the processes of transcription, post-transcriptional processing and protein synthesis (Beckwith & Yanovsky, 2014). Thus, the analysis of circadian pattern of proteome is useful in analyzing how many fold transcriptional and translational steps vary to maximize organismal adjustments over day and night.

Our study revealed an integrated pattern/regulation of proteome in the body of the fly, which could be necessary for optimizing growth and fitness. In this study, observation of multiple hits for single protein spot could be due to (i) very close-localization of two different spots of proteins, (ii) isoforms of the same proteins with a very close mass and pI and (iii) post-translational modifications. In some cases, a considerable variability in volume contribution of protein spots was observed (e.g., 6-phosphofructokinase at 04:00—0.0168 ± 0.080 or enolase at 16:00—0.1206 ± 0.1163). This could be owing to minuscule variations in the pI of proteins, their posttranslational modifications (like phosphorylation) and presence of isoforms. The missing spots at certain time points (Figs. 1A–1F) could indicate a circadian variation in the expression of proteins.

The gene ontology (GO) analysis proves beneficial in the identification of most meaningful functional aspects occurring in a given set of related gene products or biological insights into the system being studied especially when involving large proteome catalogs, like those that were generated via quantitative proteomics (e.g., ITRAQ and SILAC). Since, the focus of the current study was to investigate the overall pattern of temporal variation as well as to document the rhythmic build-up of proteins in *D. melanogaster* via 2-DE/MS (qualitative proteomics), the study greatly emphasizes on the correctness and the depth of analysis. We have therefore, presented the results of the experiment typically as a list of proteins (Al-Obaidi et al., 2014; Jessie et al., 2014). Generic biological processes annotated in this study (Table 1) were categorized based on the GO annotation found at the Uniprot Knowledgebase (UniProtKB).

The PERIOD protein is expressed only during daytime in neurons and tissues and is absent during nighttime (Plautz et al., 1997a; Plautz et al., 1997b). In our study, in the whole fly homogenate, we could not identify the protein. The reason may owe to the sensitivity of the techniques employed (2DE/MS/MS). We think that a combination of high-performance liquid chromatography and 2DE/MS/MS would have higher sensitivity to analyse the temporal variation of the PERIOD protein in the whole fly homogenate. As our temporally oscillating proteins are involved in metabolism, muscle activity, cellular transport, protein synthesis, apoptosis and development, a clock regulated release of various neurotransmitters regulating these functions (Moller et al., 2010) could also be suggested. Numerous genes involved in carbohydrate and amino acid metabolism including enzymes and membrane transporters were reported to be rhythmic (Akhtar et al., 2002). The present results showing diurnal upregulation of main enzymes of carbohydrate/amino acid metabolism (fructose bisphosphate aldolase, arginine kinase, ATP synthase subunit *β*, vacuolar ATP synthase subunit B, arginine kinase, phosphoglycerate kinase and thioredoxin reductase I) demonstrate synchronous activation and inhibition of the pathways involved. In addition, the minimal levels of expression of numerous proteins at midnight suggest that several cellular, biochemical and physiological activities were low at night in the fruit fly. These 24-h variations may be an indirect outcome of circadian control of ingestion or under a direct circadian control, mediated by neural and endocrine entities from the master clock that is located in lateral neurons of the fly (Akhtar et al., 2002).

Of late, an extensive interconnection has been documented between the molecular circadian clock and the underlying biochemical pathways that regulate the bioenergetics of the organism. The scope includes the regulatory role played by coenzymes (NAD(P)+/NAD(P)H), reactive oxygen species (superoxide anion and hydrogen peroxide), antioxidants, and physiological events that modulate the redox state (feeding condition and circadian rhythms) in determining the timing capacity of the molecular circadian clock. Both the circadian timing system and the metabolic network are tightly interlinked (Mendez et al., 2015). In addition, circadian clock gene transcription factors in metabolic tissues synchronize metabolic fuel utilization and storage with alternating durations of feeding and fasting parallel to the rest–activity cycle (Peek et al., 2015).

Recent evidences suggest the temporal accrual of yolk proteins in the seminal vesicles of *D. melanogaster* (Majewska et al., 2014). While the mechanisms of input pathways to the central circadian clock and the core circadian clock (lateral neurons in *D. melanogaster*) are extensively known, the processes that regulate the circadian output pathways (which result in the circadian proteome profile) are poorly understood. Recent genome-wide studies in many organisms suggested extensive translational regulation by the circadian clock could mainly contribute to the temporal protein profile, despite the robust mRNA rhythms observed (Montenegro-Montero & Larrondo, 2016).

Previous studies, demonstrated via distinct oscillations of mRNA and protein synthesis of genes, have shown that several genes encoding the cytoskeleton components are under clock control (Akhtar et al., 2002). Among the 23 fast skeletal muscle myosin genes, *myh_tc*, *myh_n1*, *myh_n4*, *myo18a_2*, and *myo18b_2* showed circadian rhythmic expression and possess many circadian-related transcription factor-binding sites (Creb, Mef2 and E-box motifs) within their recognized promoter regions. In addition, the circadian expression of these 5 *myosin* genes was robustly correlated with the transcription pattern of clock genes in fast skeletal muscle (Lazado et al., 2014). Murphy et al. (2014) reported significant interaction between circadian time and exercise for muscle genes *MYF6, UCP3, MYOD1* and *PDK4*. Hence, the circadian expression of a set of muscle-related proteins in *D. melanogaster* is expected.

As the proteome of the whole fly temporally vary in multiple biological processes including metabolism, muscle activities, cellular transport, apoptosis etc., our results generally indicate that a wide range of physiological/cellular processes are fine-tuned by the rhythmic expression of protein profiles. Although, the tissue specific expression of proteins and the coordination of protein regulation in various tissues of the fly could not be analyzed in this study, potential avenues of future research in the temporal regulation of intracellular localization of proteins and, exploration of rhythmically varying proteins in specific tissue types are wide open.

## Supplemental Information

10.7717/peerj.2080/supp-1 Table S1 Supplemental Table 1 Percentage of volume contribution of protein spots/clusters of *Drosophila melanogaster.*

## Additional Information and Declarations

### Data Availability

The following information was supplied regarding data availability: The raw data has been supplied as Table S1.
